# Issues and challenges of Fischer–Tropsch synthesis catalysts

**DOI:** 10.3389/fchem.2024.1462503

**Published:** 2024-09-11

**Authors:** Muhammad Amin, Muhammad Usman, Tatinaidu Kella, Wasim Ullah Khan, Imtiaz Afzal Khan, Kang Hoon Lee

**Affiliations:** ^1^ Interdisciplinary Research Centre for Hydrogen Technologies and Carbon Management (IRC-HTCM), King Fahd University of Petroleum and Minerals (KFUPM), Dhahran, Saudi Arabia; ^2^ Chemical and Materials Engineering Department, Faculty of Engineering, King Abdulaziz University, Jeddah, Saudi Arabia; ^3^ Interdisciplinary Research Center for Refining and Advanced Chemicals, King Fahd University of Petroleum and Minerals, Dhahran, Saudi Arabia; ^4^ Interdisciplinary Research Center for Membranes and Water Security, King Fahd University of Petroleum and Minerals (KFUPM), Dhahran, Saudi Arabia; ^5^ Department of Energy and Environmental Engineering, The Catholic University of Korea, Bucheon-si, Republic of Korea

**Keywords:** Fischer–Tropsch synthesis, catalyst, syngas, hydrocarbon production, liquid fuels

## Abstract

Depletion of oil and gas resources is a major concern for researchers and the global community. Researchers are trying to develop a way to overcome these issues using the Fischer–Tropsch synthesis (FTS) process. The FTS reaction converts a mixture of hydrogen and carbon monoxide gases into a liquid fuel. The reactions are performed in the reactor and in the presence of a catalyst. A series of catalysts, such as iron, cobalt, nickel, and ruthenium, have been used for the FTS process. In iron-based catalysts, the Fe_5_C phase is the active phase that produces C_5+_ hydrocarbons. At higher conversion rates, the presence of water in the products is a problem for cobalt catalysts because it can trigger catalyst deactivation mechanisms. Ni-based catalysts play key roles as base catalysts, promoters, and photothermal catalysts in FTS reactions to produce different useful hydrocarbons. Ruthenium catalysts offer not only high activity but also selectivity toward long-chain hydrocarbons. Moreover, depending on the Ru particle size and interaction with the oxide support, the catalyst properties can be tuned to enhance the catalytic activity during FTS. The detailed reaction pathways based on catalyst properties are explained in this article. This review article describes the issues and challenges associated with catalysts used for the FTS process.

## 1 Introduction

Energy consumption is rapidly increasing due to industrialization. Fuel is essential for industrial applications. Renewable and non-renewable energy sources are the two major sources of fuel (Amin et al., 2022b). Renewable energy sources have a lower environmental impact than non-renewable energy sources. However, renewable energy sources, such as solar, wind, hydro, and nuclear energy, contribute only 19% of the world’s energy production. The remaining 81% of energy production comes from non-renewable sources, such as oil, gas, and coal (Bice, 2023). Oil and gas resources are rapidly depleting, which is why researchers have been working on converting coal into hydrocarbon products (Shah et al., 2023). Coal is a major source of syngas (a mixture of hydrogen and carbon monoxide).

Fischer–Tropsch synthesis (FTS) is a process that converts syngas into useful hydrocarbon products. The syngas reaction is carried out in the presence of a catalyst in the reactor (Adnan et al., 2021). A series of reactors, including fixed-bed reactors, slurry bubble columns, tubular reactors, and fluidized bed reactors, were used for the FTS process. Many research articles have been published on FTS catalytic systems using different transition metal catalysts based on the targeted products. In addition, a series of catalysts, such as iron, cobalt, nickel, and ruthenium, have been used for FTS for a long time (Liu et al., 2022; Amin et al., 2024). Among all metals, iron, cobalt, nickel, and ruthenium exhibited adequate activity in the conversion of syngas to hydrocarbons and oxygenates, attributed to their higher hydrogenation activity (Torres Galvis and De Jong, 2013). First-row transition metals, e.g., Fe, Co, and Ni, can operate as a source or sink for electrons, as well as exchange electrons with other species, exist in multiple oxidation states, and undergo redox reactions (Edla et al., 2016; Zhou and Zhou, 2016; Popat et al., 2019). Therefore, compounds of these transition metals are used as catalysts in FTS.

Iron-based catalysts are cost-effective and offer flexible product selectivity. However, they are highly active in the water–gas shift (WGS) reactions. They are commonly utilized at low H_2_/CO ratios and are susceptible to carbon deposition and deactivation at high reaction temperatures. Cobalt-based catalysts, particularly metal-oxide-supported cobalt catalysts, have gained significant interest because of their exceptional intrinsic activity, high chain-growth probability, and low activity toward the WGS reaction (Gupta et al., 2023). The features of Co-based materials depend on their geometrical morphology, surface composition, and metal–support interaction (MSI). At higher conversion rates, the presence of water in the products is a problem for cobalt catalysts because it can trigger catalyst deactivation mechanisms. Hydrothermal sintering, the oxidation of cobalt metal to cobalt oxide, and the presence of irremediable cobalt-support compounds are some of these mechanisms (Van de Loosdrecht et al., 2013). Other important considerations include the size of the cobalt nanoparticles, support material, and catalyst synthesis process. Nickel is the fourth most abundant transition metal on the Earth and one of the most frequently utilized elements in metal-based catalysts. Ni is more active in hydrogenation and reforming reactions (De et al., 2016). Nobel Laureate Paul Sabatier described Ni catalysts as “Ni can do all kinds of work and maintains its activity for longer periods” by changing catalyst preparation conditions (Enger and Holmen, 2012a). Ni-based heterogeneous catalysts have been used for CO and CO_2_ hydrogenation processes (Usman et al., 2024). Particularly in FTS, Ni catalysts play a vital role in producing different ranges of hydrocarbons and oxygenates, and their performance depends on the Ni nanoparticle size, morphological optimization, and exploration of novel bimetallic combinations on suitable supports. Among FTS catalysts other than transition metals, ruthenium-based catalysts have shown promising activity, higher stability, and a lower extent of deactivation (Abbasi et al., 2019; Badoga et al., 2020). The high activity performance of these catalysts facilitates FTS operation at relatively lower temperatures compared to other types of catalysts (∼180°C) (Davis, 2009; Jiang et al., 2017a; Chun et al., 2020; Chen et al., 2022).

Every catalyst has its own advantages and disadvantages in the FTS. In this review, we discuss the issues and challenges of Fe-, Ni-, Co-, and Ru-based catalysts used for the FTS process.

## 2 Fe-based catalysts for FTS

Iron catalysts have been used in the FTS process because they provide high activity and olefin selectivity. Furthermore, iron catalysts are classified into bulk and supported catalysts. In recent years, research interests have shifted from bulk to supported iron catalysts. The supported iron catalysts have plenty of advantages, such as proper iron dispersion on the surface of the catalyst, high mechanical resistance, higher surface area, and more effective use of the active phase and promoter. The catalytic performance of iron-based catalysts can be enhanced by adding the promoter. A series of promoters such as K, Sb, Au, and Ag have been used for iron-supported catalysts. The potassium promoter has a great impact on hydrocarbon production in terms of selectivity. However, some of the promoters or the presence of a small amount of sulfur or sodium in the catalyst could shift the selectivity toward short-chain hydrocarbon production (C_2_–C_4_) (Chernyak et al., 2020; Di et al., 2020; Nanduri and Mills, 2020; Li et al., 2021a; Lu et al., 2021b).

**FIGURE 1:**
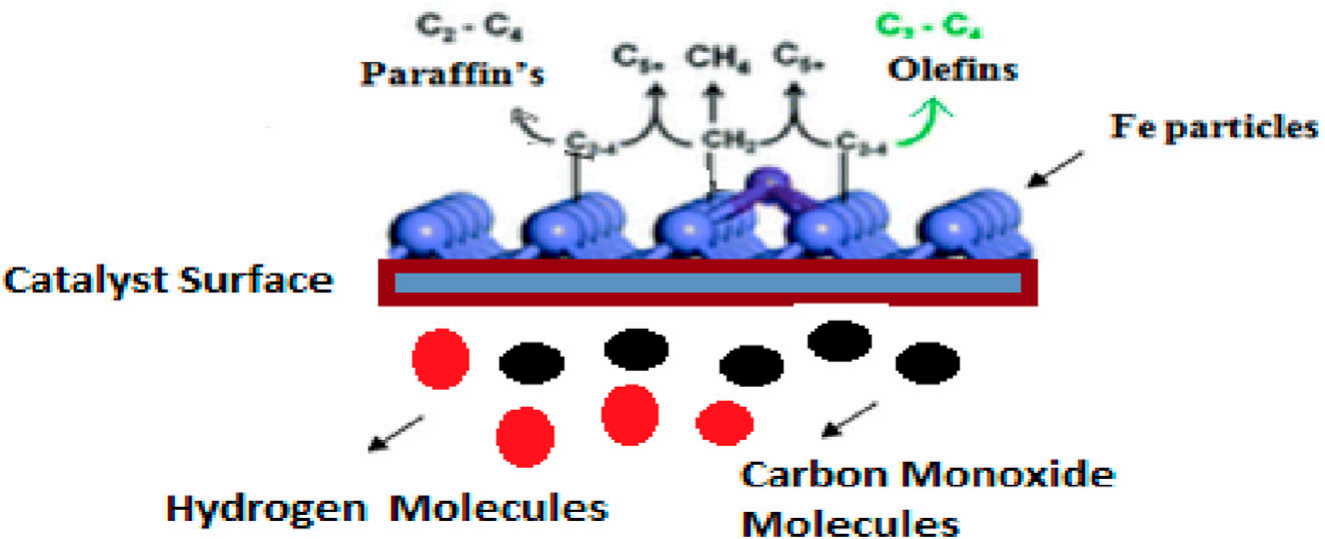
Mechanism of iron-based FTS catalysts.

The researchers improved the catalytic performance of iron-based catalysts by adding porous support or structural promoters such as SiO_2_, TiO_2_, Al_2_O_3_, and ZnO. These supports help increase the dispersion of iron particles and reduce the deactivation rate. In addition, the formation of mixed oxides such as Fe-silicate titanates is hardly reducible and non-active for FTS. The adsorption behavior of FTS products can be changed with the modification of the hydrophobic or hydrophilic properties of catalysts. In recent research, some of the researchers used perfluorodecyltriethoxysilane (PFTS) as an amphiphobic material that improves the stability of the catalyst (Fu et al., 2021; Krzyszczak et al., 2021; Lu et al., 2021a; Ma et al., 2021b; Song et al., 2021; Amin et al., 2022a; Nayak et al., 2022). Figure 1 shows the mechanism of iron-based FTS catalysts.

### 2.1 Major challenges

Fe-based catalysts are considered cheap as compared to other FTS catalysts, with a comparable syngas production rate (Amin et al., 2022a). Syngas from biomass contains H_2_S, HCl, and volatile metals. The purity of syngas is critical since a lack of purity has a detrimental impact on the reactivity. It reacts with Fe at high temperatures, producing FeS and FeCl_3_. The production of FeS results in catalyst poisoning in a reactor. The formation of the FeCl_3_ blocks the pores of the catalyst, which reduces the surface area of the catalyst. The lower surface area reduces the activity of the catalyst (Einemann et al., 2024). Mainly, in an iron-containing catalyst, the Fe_5_C_2_ phase is more active than the Fe_3_C phase (Ma et al., 2021b). The stability of the iron-based catalyst is sensitive due to the facet of iron carbides. Mechanistic investigations reveal that the increased FTS activity of {202} χ-Fe_5_C_2_ surfaces is due to hydrogen-assisted CO dissociation, which decreases the activation energy relative to direct CO dissociation over {112} surfaces. The synthesis of uniformly exposed surfaces on χ-Fe_5_C_2_ nanocrystals remains a difficult task due to the poor symmetry of the lattice structure (Wu et al., 2024).

Various kinds of support materials such as SiO_2_, zeolites, activated carbons, and carbon nanotubes have been used for the dispersion of Fe particles to increase the exposed metal sites for catalytic reaction (Yahyazadeh et al., 2022). However, for the formation of an active iron carbide phase, the surface of the carbon-based support catalyst needs to be modified. The modification leads to a tunable interaction between iron oxides and supports (Chen et al., 2021).

In order to promote the catalytic activity, Fe is often promoted with alkali metals. It provides a higher number of olefins, and a lower amount of methane is formed. Various kinds of promoters, such as K, Ce, Si, and Cu, have been used for the FTS process (Nie et al., 2019). The potassium promoter stabilized the iron carbide formation during the CO_2_ hydrogenation process, which enhanced the C_5+_ production (Martinelli et al., 2020a). However, addition of Li, Cs, K, Rb, and Ru slightly decreases the surface area of the catalyst (Uykun Mangaloğlu et al., 2018). The higher loading of the potassium promoter leads to a lower catalytic activity (Jiang et al., 2017a). The promoter may affect the crystal structure of the catalyst, reduction, and carburization process. For example, the researchers employed sodium as a promoter and found that increased loading decreases CO conversion (Buthelezi et al., 2024). During the first stage of the FTS reaction, the sodium promoter alters the electrical characteristics of the Fe-based catalyst, affecting the rate of iron carbide production (Wang et al., 2024). Therefore, the addition of a promoter has a great impact on the catalyst activity (Uykun Mangaloğlu et al., 2018). The efficiency of the promoter depends on the amount of the promoter in the active phase (Horáček, 2020). The selectivity of CO_2_ changes in the presence of an iron-based catalyst because it involves the water–gas shift process (Keunecke et al., 2024). To achieve the desired selectivity, the alkali-type promoter and loading need to be optimized (Martinelli et al., 2020a). When the catalyst is evaluated in a slurry bubble column reactor, iron-based catalysts show low resistance to attrition. Low FTS response performance is brought on by inadequate attrition resistance (Buthelezi et al., 2024). Reactor abrasion is an issue for the iron-based catalyst (Keunecke et al., 2024).

## 3 Cobalt catalyst for FTS

**FIGURE 2:**
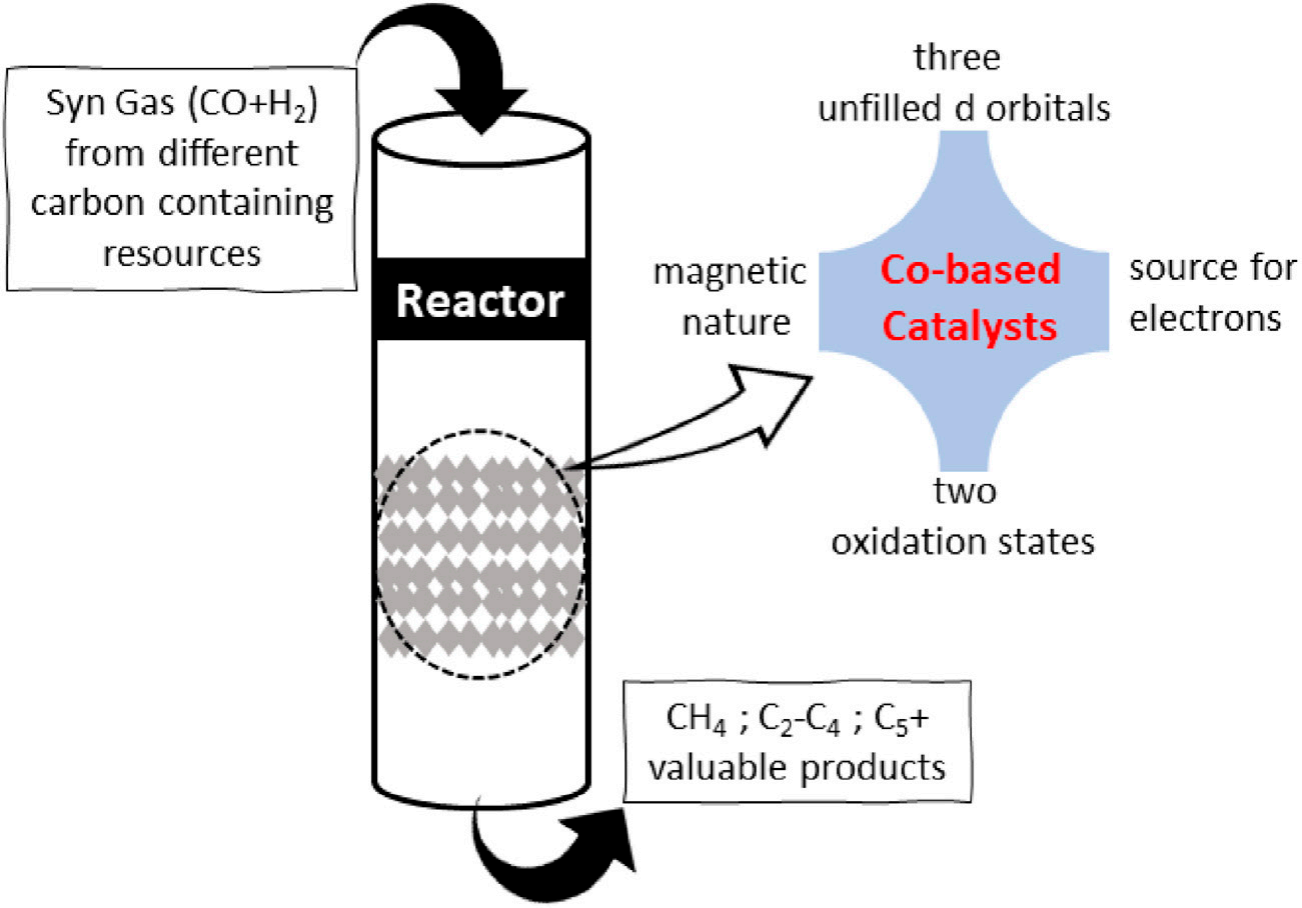
Cobalt properties for suitable FTS catalyst.

Cobalt (Co) is one of the most studied and utilized transition metals across the board in catalytic applications. Co-based catalysts have seen unprecedented growth in demand in the environment and energy-related sectors for the last few decades, causing the European Union to include them on its list of key raw materials. Co has three empty d-orbitals, whereas nickel and iron have two and four orbitals, respectively. Co forms bonds with incoming chemical species that are neither too strong nor too weak, facilitating the efficient uptake and release of reactants and products (Adeleke et al., 2020). Co has high catalytic activity because its d-orbital is only half-filled. Cobalt occurs in the Co^2+^ and Co^3+^ oxidation states in addition to its elemental form, which facilitates the formation of composites by combining with other elements or supports (Figure 2). The amount of the most stable cobalt oxide (Co_3_O_4_) can be adjusted by manipulating its redox state, as it consists of two oxidation states, Co^2+^ and Co^3+^. The ability of cobalt to conduct redox reactions (Co^2+^ to Co^3+^) makes it an excellent reagent for complex formation since it can provide electrons if the transition state of the process requires them. On the contrary, due to availability of two oxidation states, Co can catch extra electron density if surplus electrons have accumulated during the reaction (Alex et al., 2020). As a result, cobalt species can catalyze reactions involving multiple ions by reacting to generate various ions (Gupta et al., 2023).

Under Fischer–Tropsch reaction conditions, the active phase is not only metallic Co. Still, Co oxides (Qi et al., 2020), carbides of Co, carbon-deposited Co (van Ravenhorst et al., 2018), and Co-support edges (Melaet et al., 2014; Gao et al., 2020) are catalytically active as well. Co oxides are obtained by a calcination process, which is standard practice for synthesizing Co catalysts. However, due to the widespread belief that metallic Co is the premium active phase, Co calcinated samples are reduced or activated in H_2_ at high temperatures (Ellis et al., 2019; Wolf et al., 2020).

Co functions at temperatures between 200°C and 250°C, mostly producing linear paraffins (Ten Have and Weckhuysen, 2021). Cobalt’s low WGS activity means it works best with feedstocks that have H_2_/CO = 2, like natural gas. The production of linear alkanes having higher molecular weights and the creation of diesel fuel have been prioritized in the research and development of catalysts for FTS. Co shows strong catalytic activity in the synthesis of paraffin and olefin, in contrast to the predominant products of Fe-based FT catalysts, which are long-chain olefins (Zhai et al., 2021; Ribun et al., 2023).

Improved experimental technology and ongoing research have led to the design and production of many high-performing Co-based built-up catalysts. Some have been approved for industrial usage and are in the pilot stage of acceptance. According to reports, Sasol, a global chemical and energy firm, utilized a commercial Co-based catalyst. Using a Co-based catalyst supported on silica with minimal Zr promoter, Royal Shell company (Netherlands) obtained 75% conversion of syngas (CO + H_2_) and 82% liquid product selectivity. Alumina- and silica-supported Co catalyst prepared by “Synthroleum” have also shown industrial applications (Suo et al., 2022a).

### 3.1 Major challenges

The main advantage of oxide-supported Co-based catalysts is the high activity of C-C coupling in the FTS process, making them good candidates for direct conversion of CO to C_2+_ hydrocarbons (Scarfiello et al., 2023). However, the growth of metal crystallites or nanocrystals because of the relocation of metal atoms or clusters driven by thermal energy causes sintering at near 400°C (Moodley et al., 2020). The main disadvantage of oxide-supported Co-based catalysts is the weak CO_2_ adsorption and RWGS thermodynamic constraints leading to the CH_4_ production and lower hydrocarbons (Van Ravenhorst et al., 2021). Challenges of these catalysts are clarification of the function of CoO and interface between Co^0^ and CoO during CO_2_-FTS, the possible formation of Co carbide and its effects on CO_2_ hydrogenation (Scarfiello et al., 2023). Deactivation is also a key issue with oxide-supported Co-based catalysts. Poisoning, synthesis of support compounds, oxidation, coke deposition, carbide formation, and sintering deactivate Co catalysts supported on oxides. Understanding the sintering mechanism can significantly reduce the sintering rate and improve performance (Rahmati et al., 2020).

Bimetallic Co-based catalysts enhance CO_2_ conversion and C_2_+ hydrocarbon selectivity over monometallic catalysts. Traditional FTS can boost CO conversion with cobalt, and bimetallic Fe-Co catalysts allow the CO intermediate to spillover from Fe_3_O_4_ to cobalt sites, allowing CO conversion on both Co and Fe_5_C_2_ sites (Jiang et al., 2018). However, their synthesis and characterization are more challenging than those of monometallic Co catalysts. Additionally, the addition of a second metal can alter the electronic properties and surface chemistry of the catalyst, which affects its performance and selectivity (Wolf et al., 2021). The primary challenge in bimetallic Co catalysts is to control the distribution and interaction between the two metals, as it has a major impact on the catalyst’s performance (van Helden et al., 2020a).

Reducibility of Co catalysts can be increased by addition of a little amount of noble metals (Pt, Ru, Re, and Ag), via forming a larger metallic Co surface on the catalysts, which allows FT reactions to happen at a lower temperature and can significantly upsurge the CO hydrogenation rate, as well as improve CO reactivity and C5+ selectivity (Rahmati et al., 2018; Xu et al., 2020). The addition of these promoters can boost active metal centers. Ru, a structural and electrical promoter, can change the MSI to disperse and reduce Co species. Thus, noble metal-promoted Co catalysts improve reducibility, CO hydrogenation rate, and reaction inherent activity (Suo et al., 2022a). Nevertheless, these metals are expensive, making catalyst production expensive. Additionally, the metal can poison the process if the nanoparticles fuse or become larger, causing mitigation of active sites. There have also been reports concerning the promotion of undesirable reactions for certain metals like hydrogenation or cracking that decrease the production of higher hydrocarbons (Carvalho et al., 2020). Another challenge associated with noble metal-promoted Co catalysts is the promotion of unwanted reactions, such as hydrogenation or cracking. It can reduce selectivity to long-chain hydrocarbons (Suo et al., 2022b).

Non-noble metals, such as iron and nickel, have several advantages when used as promoters for Co-based catalysts. These metals are less expensive than noble metals and can be obtained by more readily available means. In addition, adding these metals can create a strong interaction between the alloy and the support, effectively enhancing catalyst stability. Nickel, for example, can promote CoO reduction and increase stability without the need for high-temperature activation (López-Tinoco et al., 2019). N and *p* doping also improves catalyst Co particle stability and dispersion (Jacobs et al., 2002; Pedersen et al., 2018). In contrast, adding these cheap promoters can also inhibit the reduction of Co, leading to lower catalytic activity (Liu et al., 2021). The amount of the promoter used for loading should also be considered, as adding more than the optimal amount can negatively affect the CoO reducibility and reduce FTS activity and selectivity (Piao et al., 2020). Furthermore, the strong bond between metal and support by adding of Ni or Fe promotes the cracking reaction, reducing long-chain hydrocarbon selectivity (Martinelli et al., 2020b). The amount of metal promoters must be considered and controlled to maximize the selectivity. Finally, it is crucial to manage the stability of the reduced species to maintain the activity (Shafer et al., 2019).

## 4 Nickel catalyst for FTS

Nickel (Ni) catalysts play a unique role in the FTS in the production of different ranges of hydrocarbons. However, as per the literature, Fe- and Co-based catalysts are the most used in the FTS process to produce hydrocarbons (Li et al., 2013; Suo et al., 2022b). Though Fe-based catalysts showed good catalytic activity, they suffered from coke formation, leading to catalytic deactivation under operating conditions of 300°C–350°C. However, Co showed good activity toward olefins selectivity, but it also yields more amount of CO_2_ due to the dominance of the water–gas shift reaction (Li et al., 2018b). Regarding the Ni catalyst, deactivation occurred at a reaction temperature of 300°C (Li et al., 2018b). Ni is very active for hydrogenation but prone to the formation of coke compared to other active metals (Fe, Co, and Ru), which makes it unsuitable as a direct base catalyst for the FTS application. It also produces volatile carbonyls at the operating reaction conditions to lose a valuable metal during the process (Khodakov et al., 2007). However, Ni as a promoter has been used for Fe- and Co-based catalysts to facilitate the dispersion of the metals, thereby enhancing their activity in the FTS reaction (Feyzi and Akbar Mirzaei, 2010; Rytter et al., 2010; Enger and Holmen, 2012b; López-Tinoco et al., 2020; Chalupka et al., 2021). In addition, Ni can also be replaced over high-cost Ru as a promoter to Co catalysts for better reduction and higher activity (Rytter et al., 2010; Enger and Holmen, 2012b). The catalytic performance in FTS systems was improved over Ni-promoted Co-based catalysts. The influence of Co/Ni ratios on TiO_2_, Nb_2_O_5_, and α-Al_2_O_3_ supports was studied, and the stable activity and selectivity for long-chain hydrocarbons (C_5+_) were attained over these Ni-promoted catalysts (Hernández Mejía et al., 2020).

During the FTS reaction, Ni-based catalysts yield more methane than other desired hydrocarbons. From this perspective, Ni as a base catalyst is more active for methane formation from syngas. In countries (China, Japan, Germany, etc.) where natural gas is not readily available, the utilization of syngas derived from coal, biomass, organic waste, and CO_2_ is a viable alternative option as a feedstock for producing synthetic natural gas. Ni, serving as a base catalyst, has demonstrated activity in the synthesis of synthetic natural gas (SNG) from syngas (Enger and Holmen, 2012b; Fan et al., 2014; Gao et al., 2015; Gao et al., 2016; Wang et al., 2016; Wind et al., 2016). Owing to the exothermic characteristics of the CO methanation reaction, the activity is quite lower over that of pure Ni-based catalysts at the lower reaction temperatures. Recently, La-promoted Ni/MgAl_2_O_4_ catalysts exhibited low-temperature activity and high-temperature stability, attributed to smaller Ni particle size, contributing to low-temperature activity, and stronger metal-support interactions, ensuring high-temperature stability (Liu et al., 2020a).

**FIGURE 3:**
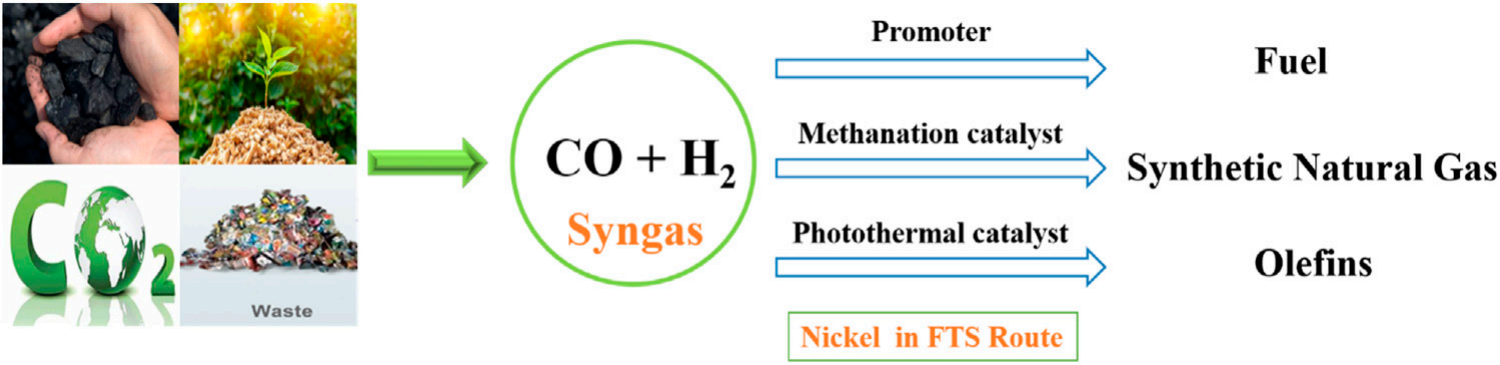
Role of the Ni catalyst in the FTS route to produce different hydrocarbons.

Moreover, the process of converting carbon monoxide (CO) into long-chain hydrocarbons requires significant thermal energy obtained from fossil fuel-derived sources, leading to the release of carbon dioxide (CO_2_). In general, CO activation and C–C coupling reactions are required to perform at high reaction temperatures (200°C–400°C) and pressures (2–5 MPa) (Li et al., 2021b). Solar-driven FTS has significant advantages in contrast to conventional FTS, which relies on substantial non-renewable energy inputs (Li et al., 2018b; Wang et al., 2020). The photothermal FTS reaction can utilize solar energy efficiently and tailor the pathways to produce value-added products. Because Ni as an FTS catalyst showed lower activity to yield long-chain hydrocarbons and produces more methane by the methanation reaction, Ni-based photothermal catalysts have been used for FTS reactions to produce C_2+_ hydrocarbons (Wang et al., 2020). The addition of non-metal atoms like O, S, and N, alters the electronic properties of the metal transition nanoparticles and results in good catalytic behavior. However, the phosphidation of TiO_2_-supported Ni nanoparticles with transition metal phosphides enhanced the catalytic activity. Titania-supported Ni_2_P/Ni catalysts were tested for the photothermal FTS reaction and showed higher selectivity toward C_2+_ hydrocarbons with lower CO conversion (Li et al., 2021b). MnO-supported Ni catalysts were reduced at different temperature ranges (250°C–600°C) and tested for the photothermal FTS reaction. Among all, the Ni-500 (reduced at 500°C) catalyst was reported with 33% olefin selectivity under UV-light irradiation (Wang et al., 2020). Double-layered NiO_x_-supported Ni nanoparticles reduced at optimized reduction temperature showed 67.0% selectivity for C_2+_ hydrocarbons and 20.9% conversion achieved due to enhanced C-C coupling reactions over the methanation reaction (Zhao et al., 2016). Ni as a base catalyst, promoter, methanation catalyst, and also as a photothermal catalyst exhibited significant catalytic performance in the CO hydrogenation reaction (FTS route) to produce a different range of hydrocarbons (methane, C_2+_ hydrocarbons, and fuel range hydrocarbons) by tuning their electronic properties and supported with suitable supports (Enger and Holmen, 2012b; Ananikov, 2015; Li et al., 2018b; Li et al., 2021b; Chalupka et al., 2021). Figure 3 shows the role of Ni catalysts in the FTS route to produce different hydrocarbons.

### 4.1 Major challenges

Ni as a base catalyst in the FTS process yields more saturated hydrocarbons and less emphasis on olefins and other longer-chain hydrocarbons due to their higher hydrogenation affinity. Though Ni has C-C coupling reactivity, the higher hydrogenation property of Ni must be controlled by tuning the electronic structure of Ni^0^ to prevent the hydrogenation of the intermediates (olefins) (Wang et al., 2020).

Ni as a promoter can be replaced over high-cost Ru as a reduction and activity promoter for Fe- and Co-based catalysts. Though the activity was good, it also formed methane due to the high hydrogenation activity. The main challenges associated with the FTS process on Ni-promoted catalysts are CO dissociation, water removal, and chain growth, which all require an optimal balance of a combination of catalytic metal surfaces. (Hernández Mejía et al., 2020). Co-Ni-based alloys showed similar activity as pure Co systems. As the Co cost is higher, it can be used with some cheaper metals (Ni) and yields similar activity. However, finding the right alloy combination and composition is very challenging. In addition, the long-term stability of the Co-Ni alloy must be studied to yield higher selectivity and activity (Van Helden et al., 2020a).

Ni as a methanation catalyst exhibited good activity due to its hydrogenation functionality. However, Ni catalysts showed low activity at lower temperatures for the exothermic nature of the CO methanation reaction due to the accumulation of heat, resulting in the deactivation of the catalyst. Maintaining higher activity at lower temperatures and preventing coke formation are challenges associated with these types of reactions over Ni-based catalysts (Liu et al., 2020b).

In the photothermal FTS system, the presence of Ni_2_P on the Ni surface favors thermodynamically occurring C-C coupling reactions, but the formation of methane and CO_2_ results in a loss of carbon atom efficiency. Therefore, the selectivity toward C_5+_ and CO conversion needs to be improved (Li et al., 2021b). Ni nanoparticles decorated on NiO_x_ support exhibited good activity for C_2+_ hydrocarbons rather than the complete formation of methane. Double-layered NiO_x_ not only absorbs visible light but also prevents methanation and enhances C-C coupling reactions when compared to the Ni metal. Controllable tuning of Ni nanoparticle formation on the doubled-layered NiO_x_ to enhance CO conversion and the higher yield of olefins and long-chain hydrocarbons are challenging tasks (Zhao et al., 2016).

## 5 Ruthenium catalyst for FTS

**FIGURE 4:**
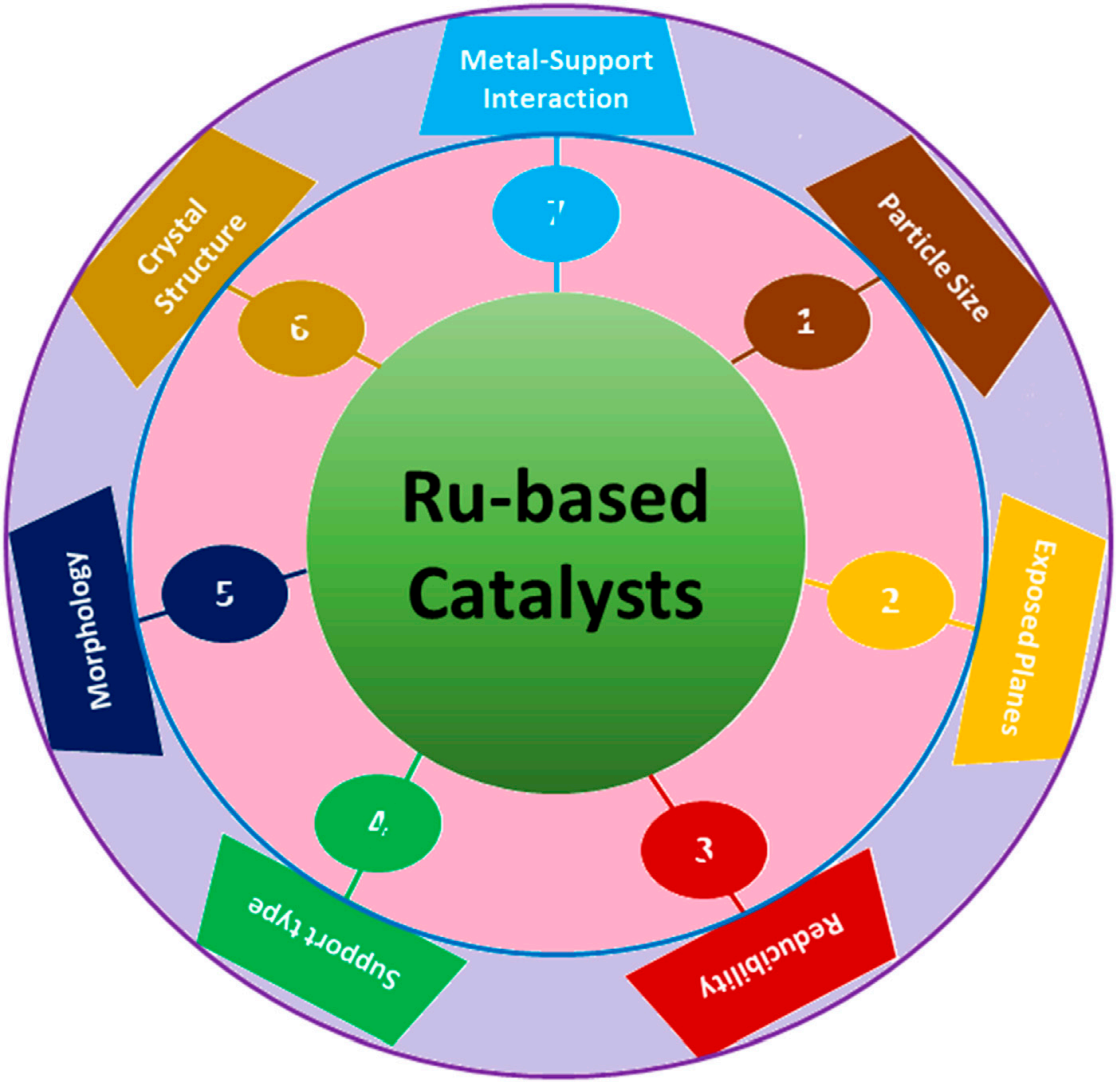
Factors affecting the performance of Ru-based catalysts during FTS.

FTS is an exceedingly efficient method for transforming syngas (a mixture of hydrogen and carbon monoxide) into essential chemicals and clean fuels (Li et al., 2018b). As mentioned earlier, the recent escalating crisis of energy has prompted extensive efforts to comprehend the essential elements of FTS, with Ru-, Fe-, and Co-based catalysts widely utilized (Hernández Mejía et al., 2018). Within these catalysts, supported Ru catalysts have exhibited promising potential for FTS due to their inherent high activity and tendency to produce long-chain hydrocarbons (C_5+_) (Zhang et al., 2020a) selectively. In general, larger particle sizes of Ru-based catalysts, approximately ∼8 nm in size, are preferred for FTS (Zhang et al., 2020a). This preference stems from the fact that smaller Ru particles tend to strongly adsorb CO, leading to a preference for methanation rather than the growth of hydrocarbon chains. Ru metal with a suitable particle size promotes CO activation and/or dissociation with or without hydrogen assistance. Hence, Ru-based catalysts have been extensively investigated for FTS to evaluate the impact of surface structure, crystal phase, particle size, and exposed planes of Ru in affecting the catalytic activity and product selectivity (Figure 4).

Various approaches have been utilized recently to improve the performance of Ru-based catalysts. For instance, Ru nanoparticles are placed inside the support to enhance the effectiveness of the catalyst during the FTS. In this regard, Ru incorporation inside the halloysite aluminosilicate nanotubes (HANTs) leads to tubular reactors that are tested for the FTS. Moreover, modification of HANTs using urea, acetone azine, and/or ethylenediaminetetraacetic acid (EDTA) promotes Ru insertion. Further treatment of this modified clay under a reductive environment facilitates a dense population of Ru nanoparticles with 3.5 nm size and 2 wt% loading. These treatments and modifications later play a vital role in the catalytic performance of these catalysts (Stavitskaya et al., 2020). Similarly, incorporating Ru nanoparticles within zeolite pores promotes controlled product selectivity, and zeolite-containing Ru particle catalyst exhibits gasoline selectivity twice that of conventional catalysts (Wang et al., 2021).

Moreover, the regulation of oxide support, e.g., TiO_2_ overlayer on Ru under variable reduction conditions, promotes the activity of Ru-based catalysts (Zhang et al., 2020b). The reduction temperature is found to influence CO activation on the oxide support overlayer at the interfaces of the supported Ru catalyst. These findings suggest that the catalyst support and its treatment are crucial in defining their performance during FTS.

The modification of Ru-based catalysts with another transition metal, such as iron and Co, also becomes critical, largely due to the interaction between two parent metals. For instance, in a supported Ru catalyst, the addition of Co doubles the activity (CO conversion); however, despite higher initial conversion, Fe addition to Ru leads to loss of activity with time (Liuzzi et al., 2021). Furthermore, Fe-modified Ru catalyst promotes oxygenate formation as well as CO_2_, while Co-modified Ru catalysts promote formation of smaller amounts of oxygenate due to smaller metal particle sizes. The surface enrichment with Ru or Co/Fe mainly controls the product selectivity.

The modification of alumina support using citric acid (CA), urea (UR), acetone azine (AA), or ethylenediaminetetraacetic acid (EDTA) for Ru- and Co-based bimetallic catalysts showed that the catalyst characteristics such as surface atom composition, metal dispersion, extent of acidic sites, and reducibility are a function of modifying agents. The CA-modified alumina-supported catalyst (Ru-Co-Al) exhibited lower activity and formation of mainly solid paraffins. EDTA modification promoted gasoline selectivity, while the AA-modified catalyst showed higher selectivity to diesel. The UR-modified catalyst remained the optimal performing catalyst with 37.3% CO conversion and C_5+_ selectivity of 79.4%. UR modification facilitated the formation of Ru-Co alloy, leading to better reducibility and moderate acidity that played a role in optimal performance of this catalyst (Mazurova et al., 2024).

### 5.1 Major challenges

The Ru size variation promotes suitable metal–support interaction that positively influences the chain growth during FTS (Zhang et al., 2020a). However, despite an increase in activity with an increase in Ru particle size, C_5+_ selectivity is reduced (Zhang et al., 2020a). Controlling the Ru particle size close to 8 nm is crucial to attain higher activity and selectivity (Zhang et al., 2020a) as larger Ru particles tend to promote undesired methanation reactions (Zhang et al., 2020a). The catalyst’s rational design at the micro level, facilitating both higher activity and desired long-chain product selectivity, remains challenging (Sun et al., 2023).

The variation in metal–support interaction (MSI) as a function of Ru size can tune the reduction behavior as well as the reactivity during FTS (Zhang et al., 2020a). Both the support type and Ru metal contents play a role in reducing the activity and product selectivity (Yan et al., 2020). Stronger MSI in the case of Ru supported on reducible oxide support such as TiO_2_ promotes surface active site enrichment (Zhang et al., 2020b). It is difficult to fine-tune the metal–zeolite bifunctional catalyst design in a nanocomposite zeolite catalyst to produce gasoline-type fuels because of the metal–acid site closeness (Přech et al., 2020).

In case of supported catalysts, the oxide support overlayer envelops Ru nanoparticles and promotes CO dissociation (Zhang et al., 2020b). In modified aluminosilicate support catalysts, the specific morphology and total acidity are major factors affecting the catalytic activity and product selectivity (Stavitskaya et al., 2020). The urea-modified aluminosilicate-supported Ru catalyst demonstrates high selectivity toward C_5+_ (Stavitskaya et al., 2020). Though the carbon aerogel-supported Ru catalyst shows higher activity, the catalyst shows no stability (Zhang et al., 2022). Furthermore, the ethylenediaminetetraacetic acid-modified aluminosilicate supported catalyst promoted the methanation reaction (Stavitskaya et al., 2020). In the case of zeolite-supported Ru catalysts, Ru nanoparticles placed inside zeolite crystals tune product selectivity (Wang et al., 2021). In spite of this, the metal encapsulated in zeolite comes out and agglomerates, leading to loss of activity (Liuzzi et al., 2021). Appropriate metal–support interaction that maintains the structure of single crystals is critical (Cheng et al., 2021).

In a promoted Ru catalyst, the role of metal–support interaction becomes prominent in comparison with the metal–promoter interaction and retaining the proximity between the promoter and Ru facilitates Lewis acidity, which, in turn, promotes CO dissociation (Eslava et al., 2020). Higher dispersion with the addition of a second metal, such as cobalt, to a Ru-based catalyst promotes activity (Liuzzi et al., 2021). Therefore, the choice of a second metal is critical in influencing the selectivity and yield of undesired products (Liuzzi et al., 2021).

The manganese promotional impact on the catalytic performance of Ru-based catalyst supported on silica revealed that manganese addition not only suppressed the formation of methane and second-stage olefin hydrogenation but also promoted activity as well as formation of long-chain olefins. Moreover, manganese addition played a role in enhancing the dispersion of Ru nanoparticles as well as the electron density of Ru active sites, leading to increased adsorption of CO followed by enhanced CO dissociation (An et al., 2023).

## 6 Future recommendations

Future studies should focus on improving the performance of iron-based catalysts such that the reduction behavior and the surface basicity of the catalysts need to be improved for higher catalyst activity and hydrocarbon selectivity. Low-cost and eco-friendly methods for the preparation of high-temperature (HT-FTS)-based iron catalysts are available, but the low-temperature (LT-FTS)-based iron catalyst needs to be addressed. The LT-FTS-based iron catalysts exhibit poor textural properties for the iron particles. It is suggested that all Co uses be strictly monitored to prevent any accidental discharge into the environment, particularly water supplies. Co toxicity, especially from used catalysts, influences living things and should be studied so that appropriate precautions can be taken. The emphasis on Ni as a catalyst for FTS reaction is still craving to address the drawbacks of this process by optimizing particle size in the nanorange and exploring different bimetallic combinations to enhance the selectivity and activity. In addition, it can be explored by integrating computational tools to design better catalyst composition efficiently. In the case of Ru-based catalysts, particle size and oxide support play a vital role in tuning the performance of the catalysts during FTS, specifically for product selectivity. The various reports on the impact of particle sizes demand further investigation on this aspect of Ru-based catalysts. Due to the contribution of factors such as support type, electronic effects, or deactivation impacts, the potential performance of Ru-based catalysts still needs to be fully evaluated, which requires to be explored in the future. The metal–support interaction manifests in various ways depending upon oxide support type, chemical composition, size of metal particles, and charge transfer. The complexity of these factors combined requires rigorous evaluation of this aspect in the FTS. In order to gain insights into the metal–support interaction at the micro level, theoretical techniques such as density functional theory (DFT) are handy. However, the disparity between experimental and theoretical data is a challenge.

## 7 Conclusion

The iron-based catalysts are cheap as compared to other FTS processes. The formation of Fe_2_C and Fe_5_C_2_ creates difficulty for the formation of the Fe_7_C_3_ phase, which is considered to be a more active phase for iron-based FTS catalysts. In the near future, we will require novel *in situ* practices to enhance our understanding of actual catalyst structures under FTS conditions. Theoretical investigations, particularly those based on artificial intelligence (AI) approaches, are proposed to understand further the nature of the available active sites and reaction mechanisms in Co-based FTS processes.

The advantage and disadvantage of Ni as an FTS catalyst include having high hydrogenation activity, which yields more methane and limits longer hydrocarbons. However, Ni can be used as a promoter and methanation catalyst and also can be used as a photothermal FTS catalyst to yield different hydrocarbons by tuning the physicochemical properties. Ru-based catalysts with a suitable particle size perform exceptionally during the FTS. The smaller particles tend to show lower activity due to site blockage on the corners/edges, along with metal agglomeration.

In comparison, larger particles close to 8 nm are ideal for the excellent performance of the catalysts. Metal–support interaction is also found to have a vital role in influencing the catalytic performance. In summary, all iron, cobalt, nickel, and ruthenium-based catalysts demonstrate key issues and challenges that are required to be addressed to achieve catalysts with better catalytic performance as well as targeting desired product selectivity.
